# Patient-Centered Outcomes Associated With a Novel Office-Based Opioid Treatment Program in a District Health Department: Mixed Methods Pilot Study

**DOI:** 10.2196/40897

**Published:** 2023-05-24

**Authors:** Theresa Coles, Hillary Chen, Andrea Des Marais, Nidhi Sachdeva, Christopher Bush, Lisa Macon Harrison, Shauna Guthrie

**Affiliations:** 1 Department of Population Health Sciences Duke University School of Medicine Durham, NC United States; 2 North Carolina Association of County Commissioners Raleigh, NC United States; 3 Aetion, Inc New York, NY United States; 4 Granville Vance Public Health Henderson and Oxford, NC United States

**Keywords:** integrated care, mental health, opioid treatment, opioid use disorder, opioid, patient-centered outcomes, public health

## Abstract

**Background:**

Granville and Vance counties have some of the highest opioid-related death rates in North Carolina, and have significant unmet needs with regard to opioid treatment. Medication for opioid use disorder (MOUD) is the most effective evidence-based approach to address opioid use disorder. Despite demonstrated efficacy and substantial need, access to MOUD is still insufficient in many parts of the United States. In order to connect patients with needed MOUD services, the district health department, Granville Vance Public Health (GVPH), established an office-based opioid treatment (OBOT) program.

**Objective:**

In this formative pilot study, we sought to describe patients’ goals and outcomes in a program delivered at a rural local health department using an integrated care approach.

**Methods:**

We used a mixed methods concurrent nested research design. The primary method of investigation was one-on-one qualitative interviews with active OBOT patients (n=7) focused on patients’ goals and perceived impacts of the program. Trained interviewers followed a semistructured interview guide developed iteratively by the study team. The secondary method was a descriptive quantitative analysis (79 patients; 1478 visits over 2.5 years) of treatment retention and patient-reported outcomes (anxiety and depression).

**Results:**

Participants in the OBOT program were 39.6 years of age on average, and 25.3% (20/79) were uninsured. The average retention in the program was 18.4 months. The proportion of individuals in the program with moderate to severe depression (Patient Health Questionnaire-9 scores ≥10) decreased between program initiation (66%, 23/35) and at the most recent assessment (34%, 11/32). In qualitative interviews, participants credited the OBOT program for reducing or stopping the use of opioids and other substances (eg, marijuana, cocaine, and benzodiazepines). Many participants noted how the program helped them manage withdrawal symptoms and cravings, which helped them feel more in control of their use. Participants also attributed improvements in quality of life to the OBOT program, such as improved relationships with loved ones, improved mental and physical health, and improved financial stability.

**Conclusions:**

Initial data show promising patient outcomes for active GVPH OBOT participants, including reduction in opioid use and improvements in quality of life. As a pilot study, a limitation of this study is a lack of a comparison group. However, this formative project demonstrates promising patient-centered outcome improvements for GVPH OBOT participants.

## Introduction

Medication for opioid use disorder (MOUD) is the most effective evidence-based approach to address opioid use disorder (OUD) [1]. MOUD entails prescribing medication, such as buprenorphine, to help patients manage their cravings and symptoms of withdrawal that occur when stopping or reducing use of opioids. There is strong evidence showing that MOUD reduces opioid use, retains patients in treatment, and saves lives [2,3].

Despite demonstrated efficacy and substantial need, access to MOUD is still insufficient in many parts of the United States [4], especially among individuals who are uninsured or underinsured. For individuals in rural areas, barriers to access are compounded even further, partially due to geographic access challenges and stigma [5,6]. Efforts are in progress to address OUD in rural Appalachia through an emergency department–based MOUD program in Jefferson County, Alabama [7]. A randomized trial to promote MOUD in emergency departments is ongoing in 4 states, including the University of North Carolina in central North Carolina (NC) [8]. Granville and Vance counties are adjacent, rural border counties located approximately 40-60 miles north of Raleigh, NC in central NC with populations of approximately 59,000 (Granville) and 44,200 (Vance) [9,10]. Granville and Vance counties have some of the highest opioid-related death rates in NC and have unmet needs for opioid treatment based on the rate of opioid-overdose deaths [11].

To connect patients with needed MOUD services, the district health department serving both Vance and Granville counties, Granville Vance Public Health (GVPH), established an office-based opioid treatment (OBOT) program in Vance County to increase local access to treatment services beginning in 2018. GVPH’s offering is unique in that OBOT services are provided through a local health department integrated into the primary care clinic rather than an OBOT-focused agency, a traditional behavioral health agency, or an opioid treatment program for higher-level care. The GVPH OBOT program follows a harm reduction model [12], which emphasizes a set of practical strategies and perspectives focused on treating individuals with respect and meeting individuals when they are on their substance-use journey with the goal of reducing harm [13].

The GVPH OBOT program developers made an intentional decision to incorporate OBOT in primary care to alleviate stigma for patients and facilitate participation, a unique model. The GVPH OBOT program offers MOUD (buprenorphine/naloxone [Suboxone] medication management), patient support for medication costs, one-on-one counseling (availability dependent on funding), weekly or twice-monthly group visits that include individual health monitoring check-ins and facilitated group discussions, infectious disease testing, and primary care services. GVPH’s OBOT program also offers wraparound service links to needed community resources, such as job support, nutrition security, childcare services, transportation, and clothing and appliance donations. Using a mixed methods approach, we describe patients’ goals and report on initial outcomes in the program from patients’ perspectives.

## Methods

### Study Design

We used a mixed methods concurrent nested approach, with the primary method being qualitative. Participants in the OBOT program were active (actively enrolled in the OBOT program) or inactive (actively enrolled in the past, but not enrolled at the time of data analysis).

### Qualitative Study

For the qualitative portion of the study, we conducted in-depth one-on-one semistructured interviews with currently enrolled patients of the GVPH OBOT program (interview guide presented in Multimedia Appendix 1). Individuals were eligible to participate if they were aged 18 years or older, and an active or previously active patient in the OBOT program.

The study design initially allotted 6 months recruiting up to 30 patients for in-person interviews at GVPH. Recruitment was extended to 8 months to accommodate the challenges of recruiting related to the COVID-19 pandemic, namely, a reduction of in-person clinic visits during the pandemic. GVPH clinic staff was trained to provide brief introductory study information to patients in the OBOT program. Patients interested in participating, filled out a web-based survey with their preferred contact information. The university study team reached out to discuss the study and answer questions. If the patient was interested in participating, then the university team scheduled an interview.

Before the interview, all participants signed a web-based consent form and verbally completed a background demographic questionnaire. Interviews lasted approximately 45-60 minutes each and were conducted using a web-based platform between June 2020 and February 2021. Trained interviewers (HC, AD, and TC) followed a semistructured interview guide developed iteratively by the study team. All interviews were recorded and transcribed for analyses.

#### Interview Content

Patient interviews covered several topics (Multimedia Appendix 1), including patients’ experiences before enrolling in GVPH’s OBOT program, expectations and goals for the program, comparisons with other programs, perceived benefits of the program, advice on how to improve the program, and unexpected benefits of the program. Topics covered in this paper include goals and expectations for the program, perceived benefits of the program, and unexpected benefits of the program.

#### Qualitative Analysis

Qualitative content analysis was used to analyze participant narratives [14]. A codebook and code definitions were developed collaboratively among Duke study team members, beginning with deductive codes and adding inductive codes after transcripts were reviewed (Multimedia Appendix 1). Duke team members used NVivo qualitative data analysis software (version 12 for Windows; Lumivero) to apply codes to transcripts. The Duke team met to code one transcript together, and then the remaining transcripts were divided among team members (TC, HC, and AD) and coded independently. To ensure consistency throughout coding, the team met regularly to discuss code applications, and one transcript was chosen randomly to be reviewed by another team member. Discrepancies were discussed, documented, and reconciled, and the codebook was reviewed and revised as needed. Throughout coding, if revisions were needed to the codebook, all transcripts were recoded to apply new codes. After coding was complete, analysts summarized findings by reviewing the code reports and transcripts in written format for each code. Themes were identified through review and discussion of codes and quotes. Descriptive statistics were calculated for the demographic and background characteristics of the qualitative participants using Excel (Microsoft Corp).

### Quantitative Study

The quantitative portion of our study focused on descriptive analysis of patient characteristics, including demographics, treatment retention, and anxiety and depression outcomes. Patient characteristics were described with frequencies and proportions for categorical variables and mean with SDs or medians with IQR for continuous measures. Quantitative data were collected by GVPH for all patients who entered the program from January 2018 through July 2020 as a standard of care. The university team retrospectively analyzed data that was captured during this time period. Depression was evaluated using the Patient Health Questionnaire (PHQ-9) [15] and anxiety was measured using the General Anxiety Disorder [16]. PHQ-9 and General Anxiety Disorder questionnaires were administered at the initial patient visit, 1 month, 3 months, and as necessary based on treatment goals. We categorized moderate to severe depression as PHQ-9 scores ≥10.

### Ethics Approval

This study was approved by the Duke Health Institutional Review Board (Pro00104650). Verbal consent was obtained before conducting all qualitative interviews. For verbal consent, study personnel provided potential participants with a description of the purpose of the project, details about the length of the interview and content discussed during the interview, possible benefits and risks associated with participating in the study, how the information will be kept confidential, the payment received, right to decline participation or withdraw from the study, and who to contact if individuals had questions. Data for the quantitative study were retrospectively collected under institutional review board (IRB) waiver of signed consent. Privacy and confidentiality were ensured in the qualitative study by storing data on secure drives that were only accessible to IRB-approved research team members. Qualitative data were deidentified for analysis and no identifying information is provided in publications. Privacy and confidentiality were ensured in the quantitative study because retrospectively collected data from GVPH were deidentified before sharing it with analysts. Qualitative participants were offered a US $30 gift card for participation. No incentives were offered to the quantitative analysis participants because the quantitative study was retrospective.

## Results

### Qualitative Study: Quality of Life Outcomes

The qualitative study included 7 actively enrolled patients (the team attempted to recruit and share study information with previously enrolled patients in the OBOT program, but were unsuccessful at enrolling these patients). Table 1 presents aggregate characteristics of patients who participated in the qualitative portion of the study.

Patients highlighted the quality of life outcomes they felt were particularly important by discussing initial goals for joining the program, successes they attributed to the program, and what they were excited about for their future. Patients often describe the program helping them “get back on track,” with discussions relating to 5 themes, which are as follows: (1) reducing or abstaining from opioids or other substances; (2) improving financial stability; (3) engaging in the workforce or training or education programs; (4) working on healthy relationships; and (5) improving mental and physical health.

**Table 1:** Characteristics of the patients who participated in the qualitative study.

Characteristic		Participants (N=7), n
**Gender**		
	Male	4
	Female	3
**Race**		
	White	6
	Black or African-American	1
	Spanish, Hispanic, or Latino origin (yes)	0
**Education**		
	Less than high school	1
	High school graduate or equivalent	3
	Completed college or graduate school	3
**Insurance type**		
	Public (Medicare or Medicaid)	5
	Private plan	1
	Uninsured	1
**In the past month, how hard has it been for you to pay for the very basics like food, housing, medical care, and heating? Would you say…**		
	Very hard or hard	3
	Somewhat hard	1
	Not very hard	3
**Is someone available to help if you need it?**		
	Never or rarely	1
	Sometimes	0
	Usually or always	6
**Do you have at least one person you can rely on in case of an emergency?**		
	Yes	7

#### Reducing or Abstaining From Opioids or Other Substances

The first theme was reducing or stopping the use of opioids or other substances; this theme was expressed by all participants and was particularly present as an initial motivation for joining the program, with many participants expressing that their main goal was to start MOUD to help manage withdrawal symptoms associated with delayed or reduced opioid use. Outside of opioids, participants also mentioned having a goal to reduce or stop their use of marijuana, cocaine, or benzodiazepines. One patient described the change they wished to see in their life related to opioid use:

Honestly, at first, there was no goal other than to not wake up in the morning and the first thing that popped into my head is I have to [use drugs]. My main goal was to be able to function during the day.

Participant 4

Participants also discussed this theme when asked about successes they attributed to being on MOUD through the program. Many noted how MOUD helped them manage withdrawal symptoms and cravings, which helped them feel more in control of their use and make decisions to stop using. Some participants also discussed the program’s positive impact on their use of other drugs like cocaine and marijuana.

It is a big thing to have something to help with the edginess of opioids, the [MOUD] really helps. It’s been definitely a lifesaver for me…And because my life has changed since I’ve been in the program, [next month] will be a year [without using opioids]…The program has definitely worked out for me.

Participant 2

#### Improving Financial Stability

Participants who discussed improving financial stability focused on obtaining a new job, owning a home, getting out of debt, or saving money and improving their finances more generally. This theme was brought up when discussing goals, successes of participants attributed to the program, and what they were excited about for the future. Many explained that not having to spend money on opioids to manage withdrawal symptoms allowed them to save more money or use their money instead on longer term goals. One participant also explained that for a period of time, the program had grant funding to cover costs associated with medication, which contributed further to their ability to save money. One participant described how not having to pay for opioids to prevent withdrawal symptoms has allowed her to save money for the longer-term goal of getting corrective dental work:

I’ve got more money in my savings than I’ve ever had in my life, and that’s thanks to getting off drugs…Fixing my teeth, that’s my main goal right now because that totally changes the way you live your life. I damaged my teeth really bad when I was on drugs, and that stuff’s really expensive to get fixed…I’m looking forward to that, and because I’m able to save some money, now that’s possible.

Participant 3

#### Engaging in the Workforce or Training or Education Programs

Many participants highlighted engaging in the workforce or in a training or educational program as an initial goal. Many were able to achieve this because of the stability MOUD provided and the support they obtained from the program. Several described starting jobs or training programs, and others expressed feeling excited about the opportunity to pursue further education or training that they would not have been able to maintain while they were managing withdrawal symptoms. One participant expressed their excitement to continue their education to fulfill a 10-year goal:

I’m looking into going back to school in the near future…which has been a goal of mine for about 10 years now…I thought about maybe going and getting some kind of certificate or something, for now, just to get my foot in the door start making money. Somewhere better than what I’m making [now]. And then going and figuring out what exactly I want to do.

Participant 1

#### Working on Healthy Relationships

Several participants identified improving relationships with friends or family as an initial goal and resulting success of the program. Some explained that with MOUD, they did not have to spend time seeking out opioids to manage symptoms of withdrawal, so they could instead spend that time with their loved ones:

… I want to get through this [and have] a normal routine… [Being with] family and friends and not having to get out and search for opiates and stuff like that. It just took so much time and effort to do that and I didn’t have time for the other things.

Participant 6

A few participants also explained how being on MOUD allowed them to make decisions to move on from relationships they felt were not helpful for their recovery and focus instead on forming positive relationships through support groups or engaging with their community through activities like team sports or church.

#### Improving Mental and Physical Health

Although some participants started the program with the goal of improving their physical or mental health, many described resulting physical or mental health improvements they attributed to their participation in the program. Participants explained how being enrolled in the OBOT program helped them establish better sleep habits; abstain from or reduce substance use; engage in healthy weight loss; quit smoking tobacco; address anxiety and depression; and feel “stable,” “peaceful,” or “more present” in their life.

One participant described improvements in their mental health and feeling a sense of control they did not feel before joining the program:

My mental health is great. I haven’t had any episodes or depression in 3 years. Sometimes I’ll have mood swings, but it’s very minor and it’s not anything like it used to be like. Sometimes I would be up for days and just go off on a shopping spree or drug spree and I had no control over my impulses and things like that. Now, I have that control and it’s just a miracle really.

Participant 7

### Quantitative Study

From January 2018 to July 2020, the GVPH OBOT program saw 79 patients, including 1478 visits (average of 18 visits per patient). The average age of participants was 40 years with a range of 24-68 years. A little more than half (43/79, 54%) were female. The majority of participants were White (65/79, 82%) and 75% (59/79) had either public or private insurance, as shown in Table 2.

From January 2018 to July 2020, there were 35 active (enrolled in the program) and 44 inactive (patients who had left the program as of July 2020) patients. Overall, 73% (58/79) of patients had completed 3 months of the OBOT program (Table 3). Among active participants (n=35), only 1 patient did not stay in the program to 6 months at the time of data collection. Of the 35 patients who made it to 3 months at the time of data collection, 10 patients (10/35, 28.6%) tested positive for opioid use using urine toxicology screening at 3 months. Of the 34 patients who were actively involved in the program for 6 months, 4 patients (4/34, 11.8%) tested positive for opioid use using urine toxicology screening at 6 months, signaling a decline in proportion of opioid use among active participants.

Among active participants, improvements in median patient-reported depression and anxiety scores were observed as patients progressed through the program (Table 4). Depression and anxiety were not assessed for individuals who did not continue to participate in the program. There were 23 individuals (23/35, 66%) in the program who had moderate to severe depression (PHQ-9 scores ≥10) at their initial assessment, and 11 at the most recent assessments (11/32, 34%), representing a median change in score of 5 points from the initial to the most recent assessment.

**Table 2:** Characteristics of Granville Vance Public Health office-based opioid treatment program participants (N=79).

Characteristic		Values
Age (years), mean (SD)		39.6 (10.9)
Female, n (%)		43 (54)
**Race (check all that apply), n (%)**		
	Black	14 (18)
	White	65 (82)
**Insurance (check all that apply), n (%)**		
	Private insurance	15 (19)
	Public insurance	44 (56)
	Uninsured	20 (25)

**Table 3:** Retention in treatment from January 2018 to July 2020 by program status.

	Active^a^ participants (N=35)	Inactive^b^ participants (N=44)	Overall participants (N=79)
Retention in treatment (months), mean (SD)	18.4 (8.1)	4.7 (5.1)	10.8 (9.5)
Retained in treatment for 3 months, n (%)	35 (100)	23 (52.3)	58 (73.4)
Retained in treatment for 6 months, n (%)	34 (97.1)	11 (25)	45 (57)

^a^Active: patients currently in the program.

^b^Inactive: patients who had left the program as of July 2020.

**Table 4:** Change in patient-reported depression and anxiety scores.

Measure name	Outcome measured	Initial assessment (n=35), median (IQR)	Most recent assessment (n=32), median (IQR)	Change (most recent-initial) (n=32), median (IQR)
Patient Health Questionnaire-9^a^	Depression	10 (6 to 19)	4 (2 to 10)	−5.0 (−10.5 to −1)
General Anxiety Disorder-7^b^	Anxiety	13 (7 to 16)	4.5 (0.5 to 10)	−5.0 (−11 to −1)

^a^Higher scores indicate worse depression.

^b^Higher scores indicate worse anxiety.

## Discussion

This study provides preliminary evidence showing promising retention outcomes and improved patient outcomes associated with enrollment in the GVPH OBOT program. In terms of retention, approximately 75% (58/79) of individuals who started the OBOT program were still enrolled in the program after 3 months, and more than 50% (45/79) of all patients who started the OBOT program were still enrolled at 6 months, exceeding definitions of successful retention [17]. The quantitative portion of the study showed depression and anxiety scores improved while patients were enrolled in the program. The qualitative portion of the study also provided initial evidence for improvements in the quality of life for patients engaged in OUD treatment. Although the desire to reduce opioid use was the primary goal for most GVPH OBOT patients, participants also described other goals and perceived outcomes, such as improving relationships and mental health, financial stability, finding a job, and addressing other health issues. Consistent with conceptual models of quality of life [18,19], OBOT programs should consider formally measuring patient-centered outcomes that are most important to patients and represent a true improvement in their quality of life and recovery. Such data may support future comparative effectiveness studies.

The primary strength of this study is the holistic lens through which patient outcomes are described, with quantitative and qualitative methods. Patient characteristics were largely similar among the quantitative and qualitative samples, except for insurance type. The similarities among both samples signal that the qualitative sample was generally representative of the patients who were enrolled in the OBOT program.

As a formative study, limitations of this study include the lack of a comparison group and limited data for tracking outcomes quantitatively. Another key limitation was the inability to conclude which aspects of the GVPH-OBOT program or GVPH more generally led to the outcomes described. MOUD is the most effective treatment for OUD, and a significant proportion of the improvements shared by participants were likely due strictly to MOUD. It is less clear what other program offerings directly influenced outcomes. Other studies provide evidence that GVPH-OBOT offerings, such as covering medication costs and counseling, improve outcomes. For example, one study found that covering medication costs, a GVPH-OBOT offering improves retention [20]. One-on-one pain counseling is associated with a reduction in cravings and opioid use [21]. Another qualitative study showed that group counseling, a GVPH-OBOT offering, elicits healthy, supportive, relationships among attendees, and supports accountability [22]. In lieu of the ability to conclude which specific aspects of the program influenced outcomes, the study team qualitatively explored what was working well in the program and what was not working well from the patient and clinician or staff perspectives. This information is publicly available in a case study [23]. Results from the case study show that clinicians and staff believed that training in harm reduction principles and a focus on building relationships with patients were key components of the perceived success of the program. Results of the case study also indicated that GVPH-OBOT participants attributed covering the cost of medication as a motivator for patients to initiate the program. Other aspects are discussed in detail in the case study. In addition, participants in the qualitative study were currently enrolled in GVPH’s program; the experiences of patients who were no longer enrolled in GVPH’s program could be different from patients who were enrolled at the time of the interviews. Patient interviews were originally planned to be conducted in person with a larger cohort of individuals, but data collection coincided with the COVID-19 pandemic and influenced our ability to recruit and connect with patients. Whether the program outperforms other program models is unknown, and more systematic data collection will be needed to compare programs with different models. Systematic data collection would also elucidate causal pathways between the GVPH program and patient outcomes.

GVPH follows an Academic Health Department Model [24], which emphasizes connection with academic partners to help evaluate and improve their services. GVPH is expanding its program to include counseling around adverse childhood experiences. This study represents 1 step forward in identifying strengths and weaknesses of the current data collection practices and opens opportunities for continued, more systematic evaluation of GVPH’s OBOT program as it evolves.

As funds become available to local municipalities from the National Opioid Settlement, opportunities exist to conduct comparative effectiveness research and natural experiments on new and existing interventions for OUD. This is a unique opportunity for researchers, clinicians, and patients to capture patient-centered outcomes and promote evidence-based interventions to improve the lives of individuals with OUD, as well as the consequent health of our communities.

### Conclusions

Preliminary data suggest that GVPH’s OBOT services provided through a local health department are associated with a reduction in patients’ opioid dependence and an improvement in quality of life. Though GVPH serves a smaller population than urban NC counties, each life affected, including family members and community members of individuals with OUD, is critically important. GVPH’s OBOT program provides a vital access point to treat OUD for individuals in Granville and Vance counties. Initial results demonstrate promising improvements in the quality of life for individuals in the GVPH OBOT program, and additional program evaluation will need to be conducted to better understand which outcome improvements can be attributed to which features of the GVPH OBOT program. Other district health departments in the United States can look toward GVPH’s OBOT program for guidance on how to develop, refine, and evaluate their MOUD programs.

## Appendix

Multimedia Appendix 1 1. Interview guide - patients. 2. Codebook.

## Abbreviations

GVPH: Granville Vance Public Health
IRB: institutional review board
MOUD: medication for opioid use disorder
NC: North Carolina
OBOT: office-based opioid treatment
OUD: opioid use disorder
PHQ-9: Patient Health Questionnaire-9

## Data Availability

The data sets generated and analyzed during this study are not publicly available due to the small sample size and concerns about patient confidentiality and identifiable data despite removable of identifiers. Data are available from the corresponding author on reasonable request.
