# Supplementary material for: Patient-Centered Outcomes Associated With a Novel Office-Based Opioid Treatment Program in a District Health Department: Mixed Methods Pilot Study
Source: JMIR Form Res. 2023 May 24;7:e40897. doi: 10.2196/40897 (PMC10248770; doi:10.2196/40897)
Supplement: Multimedia Appendix 1 [file formative_v7i1e40897_app1.docx]

**APPENDICES**

Appendix 1. Interview Guide – Patients

Appendix 2. Codebook

**Appendix 1. Interview Guide – Patients**

1. How long have you been part of the medication assisted treatment for opioid use disorder program at Granville Vance Public Health?
2. How did you hear about this program?
3. What had you heard about the program before you started it?
4. How did you get involved in this program?
   1. Was there a waitlist or period of time you had to wait to start the program after first reaching out?
   2. Did you have worries or concerns about being a part of the program before you started it? If so, what were your worries or concerns?
5. How would you describe your life before joining the program?
   1. How would you describe your substance use?
   2. How would you describe your mental health before joining the program?
   3. How would you describe your physical health before joining the program?
   4. How would you describe your relationships and connections with other people before joining the program?
   5. What was your job status before joining the program?
6. Had you participated in any other substance use programs or treatment before joining the program?
   1. Do you feel that that program/treatment helped you reach any of your goals? If so, how?
   2. How are your experiences with this program different than other programs you have participated in before?
7. What were you hoping to get out of the Granville Vance Public Health program when you started it?
   1. What were your goals?
   2. Did you think the GVPH MAT program would help you achieve those goals? Why?
   3. What did you want life to be like because of the program?
      1. Family
      2. Employment
      3. Mental health
      4. Physical health
      5. Day-to-day activities
8. Describe your experiences with your one-on-one visits.
   1. Were your one-on-ones in person, by phone, or by internet?
   2. How have the one-on-one sessions helped you achieve your goals?
   3. Were the one-on-one sessions what you expected them to be or something different? How so?
9. Have you attended any group sessions? [If yes, continue to question 9. If no, skip to question 10.]Describe your experience with the group sessions.
   1. How did you feel going to the health department for your first group session?
   2. Was the experience different than you expected? How so?
   3. How have the group sessions helped you achieve your goals?
   4. What was your impression of the staff running the group session?
   5. What was your impression of the other people participating in the group session?
   6. [If switched from group to one-on-ones due to COVID-19] How did you feel about switching from group sessions to one-on-one sessions?
10. Has the MAT program in general provided you with support for other issues besides substance use?
    1. What resources has the program connected you to?
    2. What other resources do you still need that you have not received?
    3. Was there a change in the support offered since coronavirus became a problem? If so, what changed?
11. What do you like about the program?
    1. What do you feel is most helpful about the program? Has this changed over time? If so, how? Are these changes because of coronavirus or something else?
    2. Have you tried to get others involved in the program? If no – how come? If yes - what have you told them about the program to get them interested in joining?
12. What would you change about the program?
    1. What do you wish the program offered? OR What do you wish the program did differently?
    2. How could the program be changed to better serve your needs?
13. How would you describe your life now being part of the program?
    1. How would you describe your substance use now?
    2. How would you describe your mental health now?
    3. How would you describe your physical health now?
    4. How would you describe your relationships and connections with other people in your life now?
    5. What is your work status now?
       1. Did you experience job changes or insurance changes due to coronavirus? If so, what changed?
       2. Did the job changes affect your participation in the MAT program?
    6. How did you know that the program was helping you? What changed over time?
14. What are some things you enjoy doing in your life now that you would not/could not do before joining the program? Were there changes you started enjoying before COVID, in addition to things currently?
15. How would you define being “successful” in the program?
    1. If someone wanted to know about “successes” that the program is helping you achieve, what would you tell them about?
16. What are your current goals?
    1. Do you think the program will help you reach these goals? How so?
    2. Are these goals different from the goals you started out with when you first joined the program?
    3. Have your goals changed because of COVID-19? If so, how? If not, tell me more about that.
17. Did anything good come out of the program that you did not expect? If so, what?
18. Did anything bad happen because of the program that you did not expect? If so, what?
19. What else would you like to share about the program or your experiences that you want to be sure we know?
20. What are you most proud of or excited about?

**Appendix 2. Codebook**

| **Parent code** | **Code** | **Description** |
| --- | --- | --- |
| Descriptors | Length of time in program | Length of time the interviewee has been involved in the program |
| Content codes | Before joining program | Use this as a double code to note when something occurred before the participant joined the program |
|  | After joining program | Use this as a double code to note when something occurred after the participant joined the program |
|  | Barrier | Interviewee describes something that is a challenge patients or providers experience related to the program |
|  | Facilitator | Interviewee describes something that is helpful for patients or providers experience related to the program |
|  | Word of mouth | Interviewee describes what they heard from other people about the program before they joined |
|  | Entry into program | Interviewee describes how they heard about the program and how they enrolled |
|  | Expectations for the program | Interviewee describes any concerns or worries they had about the program before joining, as well as expectations for the program before starting - could be true or untrue expectations about the program |
|  | Experiences with other SU programs | Interviewee describes their experiences with other substance use treatment programs |
|  | Personal life | Interviewee describes their life including mental and physical health and relationships before and after joining the program |
|  | Patient goals | Interviewee describes their personal goals; includes motivation for entering program |
|  | Trust/honesty quotes | Interviewee discusses the role of trust and honesty in their experience of the program or interactions with the provider |
|  | Other needed resources | Interviewee describes other resources they or patients involved in the program need access to (double code with ‘barrier’ if they/patients cannot access or ‘facilitator’ if it was beneficial that the program helped them/patients access) |
|  | Referring other people to the program | Interviewee describes what they tell other people about the program, and instances when they have referred other people to the program or tried to get others to join. |
|  | COVID impact | Interviewee describes impact on COVID on both life and program - double code with ‘telemedicine’ and ‘barrier' or 'facilitator' |
|  | Telemedicine | Interviewee discusses telemedicine in general |
|  | Behavioral health | Interviewee discusses the need for or experiences with behavioral health components of their treatment |
|  | What are you excited about? | Interviewee discusses what they are excited about in life |
|  | Definition of success | Interviewee describes what they think makes someone 'successful' in the program |
|  | Other people's stories | Stories about other people either in the program, or people who dropped out, or people who would qualify for the program |
|  | Program attribute | Interviewee describes components of the program (double code with 'barrier' or 'facilitator' for what’s working well/not working well and what someone would change); what you would write down in a protocol |
|  | Perceived impact of program | Interviewee describes their perceived impact of program on participant lives |
|  | Advice | Interviewee expresses advice they would give to other clinicians, clinics, or health departments interested in starting an OBOT program (could be advice from patient or provider) |
|  | Incorrect transcription | Any time there is an incorrect transcription that is particularly important to the meaning of the quote and overall analysis |
|  | Community partnerships | use when interviewee is talking about partnerships with other community members or organizations |
